# A prenatal diagnosis and genetics study of five pedigrees in the Chinese population with Xp22.31 microduplication

**DOI:** 10.1186/s13039-019-0461-1

**Published:** 2019-12-11

**Authors:** Jianlong Zhuang, Yuanbai Wang, Shuhong Zeng, Chunling Lv, Yiming Lin, Yuying Jiang

**Affiliations:** 1 Prenatal Diagnosis Center, Quanzhou Women’s and Children’s Hospital, Fujian Province, People’s Republic of China; 2 Zhejiang Biosan technology Co., Ltd, Zhejiang, People’s Republic of China; 3 Neonatal Disease Screening Center of Quanzhou, Quanzhou Women’s and Children’s Hospital, Fujian Province, People’s Republic of China

**Keywords:** Xp22.31, Microduplication, Polymorphism, single nucleotide, Prenatal diagnosis

## Abstract

**Background:**

Copy number variations (CNVs) can contribute to human phenotype, phenotypic diversity and disease susceptibility, while others may benign. In the current study, an attempt to investigate the pathogenicity of CNVs in chromosome Xp22.31 was explored.

**Methods:**

G-banding and SNP-array techniques were used to analyze chromosome karyotypes and CNVs in fetuses. Parents associate with five different pedigrees possessing high risk factors in pregnancy were considered with such parameters as advanced age, high risk of serological screening and ultrasound abnormalities.

**Results:**

The fetuses’ amniotic fluid karyotypes were 46, XX and those of their parents with the five pedigrees revealed no abnormalities. Here, we noticed a series of individuals with Xp22.31 duplications ranging from 534.6 kb to 1.6 Mb. It was detected through SNP array that the fetuses in Pedigree 1 and 2 had ~ 600 kb duplications in the Xp22.31 region of their X chromosomes which contained two OMIM genes, *HDHD1* (OMIM: 306480) and part of *STS* (OMIM: 300747). The fetuses of Pedigrees 3, 4 and 5 had 1.6 Mb duplication in the same chromosome which contained four OMIM genes: *HDHD1* (OMIM: 306480), *STS* (OMIM: 300747), *PNPLA4* (OMIM: 300102) and *VCX* (OMIM: 300229). The duplications in the fetuses of Pedigrees 1 and 5 were inherited from the non-phenotypic parents. Pedigrees 3 and 4 refused to perform parental verification. Finally, four of the five pedigrees continue towards pregnancy with no abnormalities being observed during followed-ups.

**Conclusion:**

Our study first showed duplications of Xp22.31 in Chinese population. Clinical and genetic investigation on five different pedigrees, we consider the duplication of these fragments as likely benign copy number variants (CNVs). We suggest that the duplications of Xp22.31 with recurrent duplication as a benign CNVs .

## Background

The Xp22.31 segment of the human X chromosome short arm is a region of high instability with frequent rearrangement [1]. Genomic instability is a feature of Xp22.31 region wherein deletions or mutations are associated with X-linked ichthyosis (OMIM 308100, XLI), a dermatologic disorder presenting with dry, scaly skin due to a deficiency of the enzyme steroid sulfatase (STS), usually arising from a mutation in the *STS* gene [2–4]. Xp22.31 duplications, the counterpart of the Xp22.31 deletion causing XLI containing the *STS* gene, are among the most frequent findings in genetic laboratories but are challenging to interpret [1, 5–8]. The unequal recombination flanking the *STS* region frequently produces the 1.6 Mb recurrent duplication contains four known genes, including *HDHD1*, *STS*, *VCX*, and *PNPLA4* [9]. At present, the research on Xp22.31 microdeletion has been relatively mature. However, the clinical significance of the Xp22.31 microduplication is still unclear and only a few studies are available on this. Indeed, some authors interpreted this duplication as being a benign variant [10, 11], whereas in others it is classified as pathogenic [1, 5–7], and others suggest that the duplication might only be interpreted as variants of unknown significance (VOUS) [8, 12]. Microduplication of Xp22.31 has been reported in the healthy general population but also been described in individuals with pathological conditions [13]. The consequences of this repetitive region are still unclear.

Our study is a first report from Chinese population carrying Xp22.31 microduplications. Four of the five pedigrees continued towards pregnancy, following which no obvious clinical abnormalities were observed.

## Materials and methods

### Subjects

Five fetuses of the five unrelated Han Chinese families were identified as carrying Xp22.31 duplication, after which their family histories were collected. All five of these were classified as Pedigreen1, 2, 3, 4 and 5; they had come to the prenatal diagnosis centre of our hospital for different reasons. The pregnancy women in the five Pedigrees denied having a related family history and did not have pregnancy complications.

Pedigree 1: A 27 year old pregnant woman, G1P0, was diagnosed with ultrasound abnormalities, described as being strong left heart echoes in her fetus and an enhanced echo of its intestine.

Pedigree 2: A 36 year old pregnant woman, G2P1, where no ultrasound abnormalities in the left nasal bone of the fetus were observed. The woman’s first child born in 2007 was a male with a normal phenotype.

Pedigree 3: Pregnant woman of 24 years, G2P1, was screened serologically for her high risk (T21: 1/176). This woman’s first child was a female and born in 2016, with neonatal pneumonia and brain softening that had been hospitalized for 53 days, and died for this after 2 to 3 months.

Pedigree 4: Pregnant woman of 32 years, G4P1, was screened serologically for her high risk (T21: 1/47). The woman’s first child was born in 2015; he is a male with a normal phenotype. The woman had experienced two spontaneous abortiond in the second months of pregnancy.

Pedigree 5: A pregnant woman of 33 years, G1P0, was diagnosed with ultrasound abnormalities; the left lateral ventricle of her fetus was widened, and its gallbladder was slightly larger, at 2.74 × 0.76 cm.

### Analysis of peripheral blood karyotype

G-banding analysis (C/NOR-banding when required) at a resolution of approximately 320~400 bands was performed on the parents’ peripheral fetal blood according to standard laboratory protocols and ISCN 2016 [14]. Around 2 ml of parental peripheral blood in the parents of the fetuses was collected in heparin tubes. The cells were inoculated into peripheral blood lymphocyte culture medium under sterile conditions, and the chromosome preparation was performed by the automatic chromosome harvesting system SinochromeChromprepII (Shanghai Lechen Biotechnology Co., Ltd.), with 20 karyotypes per case and five karyotypes analyzed.

### Amniocentesis and karyotype analysis

Around 20 ml of amniotic fluid was obtained by amniocentesis was collected and centrifuged, the supernatant were discarded and the pellet was inoculated into amniotic fluid medium. The media was incubated at 37 °C, 5% CO_2_ for 7–10 days. Amniocytes were harvested by trypsinization and chromosome preparation, and karyotype analysis was performed after staining with Giemsa stain. 30 karyotypes were taken per case and five karyotypes analyzed.

### DNA extraction and SNP-array detection

Amniotic fluid and peripheral blood DNA extraction were performed using the QIAamp DNA Blood Kit (QIAGEN, Germany), and the extraction method was followed as per the kit Handbook (www.qiagen.com). Enzyme digestion, ligation, amplification, purification, fragmentation labeling, hybridization, and scanning and analysis were performed according to Affymetrix CytoScan Assay USER GUIDE (http://www.thermofisher.com) using AffymetrixCytoScan™ 750 K chip, ClonTECH™ DNA amplification kit (Life Technologies, American), purification hybridization kit (Life Technologies, American), and all experimental results were obtained in 3 days. Results were interpreted with reference to DGV (http://dgv.tcag.ca/dgv), OMIM (https://omim.org/), DECIPHER (https://omim.org/), and Pubmed (https: //www. ncbi.nlm.nih.gov/pubmed/).

## Results

### Analysis of the chromosome karyotype of the fetuses and the parents

The conventional G-banding karyotype analysis of the fetuses and the parents in five pedigrees revealed that the fetuses’ amniotic fluid karyotypes were 46, XX and no obvious abnormalities were observed in the parents of the five pedigrees.

### The SNP array detecting results in the fetuses and parental verification

**Fig. 1:**
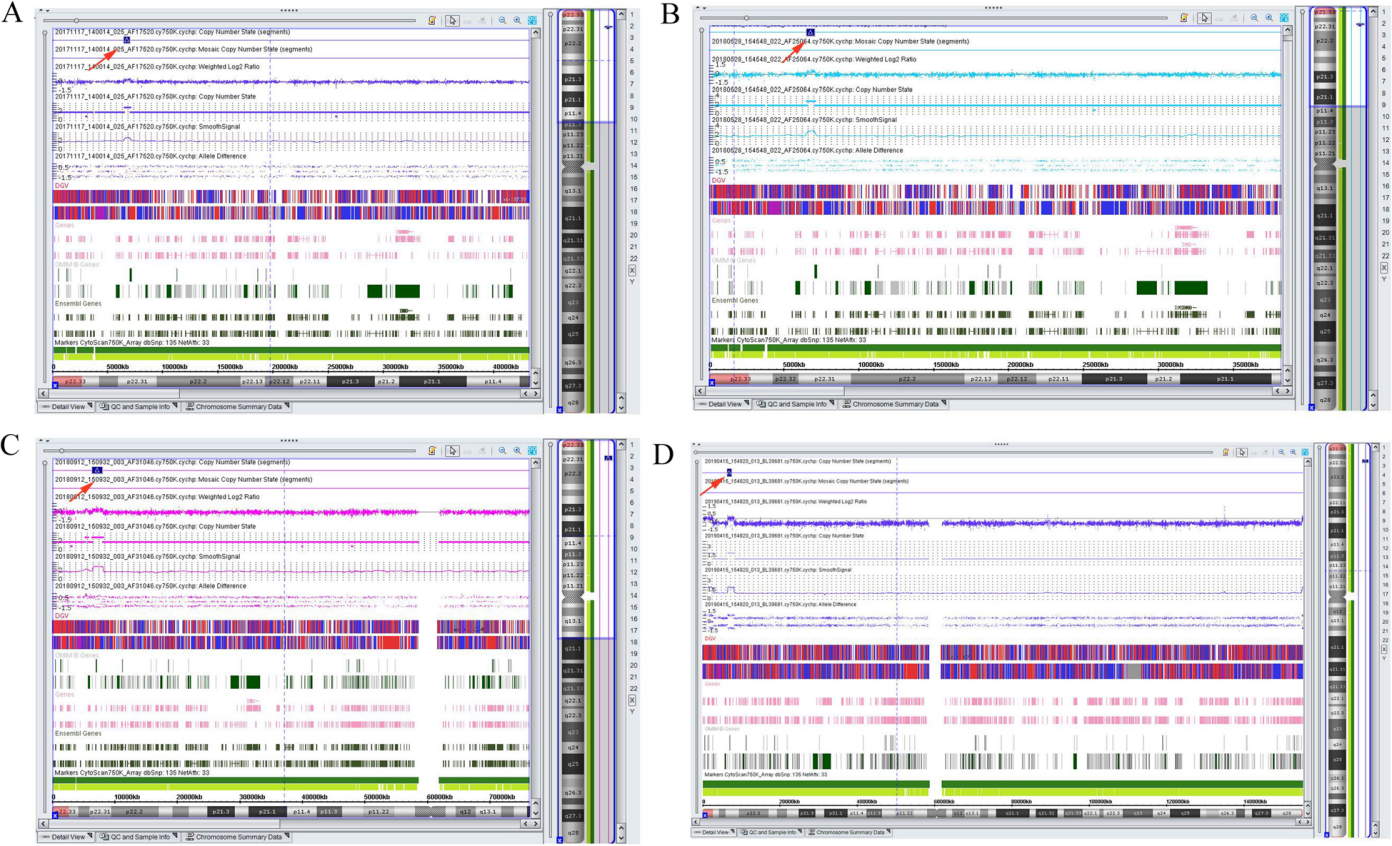
The SNP-array results for the fetuses. The red arrow indicates the position of the Xp22.31 microduplication. **a** The SNP-array detection of Pedigree 1 fetus. **b** The SNP-array detection of Pedigree 2 fetus. **c** The SNP-array detection of Pedigree 3 and 4 fetuses. **d** The SNP-array detection of Pedigree 5 fetus

**Fig. 2:**
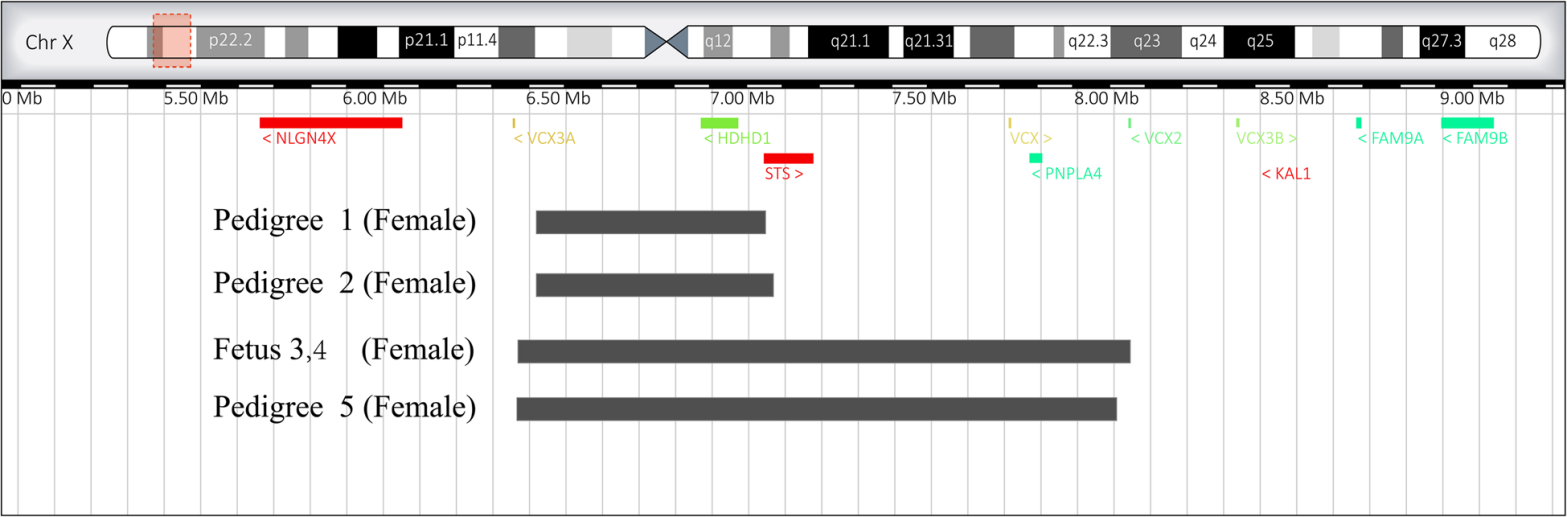
A comparison of the breakpoints of the five pedigrees. Uppermost is a schematic representation of the X chromosome was shown. The red box represents Xp22.31’s genomic location, which has been magnified below. Below, the colour bars represent OMIM genes with the gene’s names below. Beneath the genomic location bars, our pedigrees are represented with gray bars representing the sizes of the duplications

To further detect the copy number variants in the fetuses, the SNP array technique was performed. The SNP array detecting results demonstrated that the Pedigree 1 fetus had a 534.6 kb duplication in the Xp22.31 region of the X chromosome (ChrX: 6538, 033–7, 072, 640) (Figs. 1 and 2), containing two OMIM genes: *HDHD1* (OMIM: 306480) and part of *STS* (OMIM: 300747) which crossed the first exon. A 562.1kb duplication (ChrX: 6, 538, 033–7, 100, 181) was observed in the Xp22.31 region of the X chromosome in the fetus of Pedigree 2 (Figs. 1 and 2), and contained two OMIM genes: *HDHD1* (OMIM: 306480) and *STS* (OMIM: 300747) partial fragments. The duplication positions of Xp22.31 were similar in the fetuses of Pedigrees 1 and 2. In the DECIPHER database, there were several cases with intellectual and language development disability, have lesser duplications than the Pedigrees 1 and 2 (DECIPHER ID: 249205, 3,000,318, 385,446), and some cases with non-phenotype were found to possess larger fragments (DECIPHER ID: 253526, 253,523, 259,159, 253,259). In addition, the DGV database revealed a polymorphic duplication (chrX: 6, 455, 152–7, 120, 567) which was larger than the duplication of the in the two mentioned pedigrees [15]. The Pedigrees 3 and 4 fetuses had 1.6 Mb duplication region (ChrX: 6, 455, 151–8, 135, 568) in the Xp22.31 of the X chromosome (Figs. 1 and 2), which contained four OMIM genes: *HDHD1* (OMIM: 306480), *STS* (OMIM: 300747), *PNPLA4* (OMIM: 300102) and *VCX* (OMIM: 300229). The Pedigree 5 fetus had a 1.6 Mb duplication (ChrX: 6, 455, 151–8, 134, 649) and contained the same four OMIM genes as mentioned for Pedigrees 3 and 4. According to the DECIPHER database, some cases that possess a smaller duplication region demonstrate atrial septal defects, intellectual disability, autism, and other disorders (DECIPHER ID: 3782, 281,855, 281,857, 251,344), while, other cases with larger duplication fragments show non-phenotype changes (DECIPHER ID: 261600, 263,228, 308,798, 369,261). Therefore, the clinical significance of Xp22.31 microduplication is unclear. The duplications in the fetuses of Pedigrees 1, 2 and 5 were inherited from non-phenotype parents, and Pedigrees 3 and 4 refused to perform parental verification (Table 1).

**Table 1:** Chromosome microduplication position and inheritance of the five pedigrees

Pedigree	Duplication position (hg19)	Size	Fetuses chromosome karyotype	OMIM genes	Inheritance	Pathogenicity contribution
Pedigree 1	chrX:6538,033-7,072,640	534.6 kb	46, XX	*HDHD1,STS*	Inherited from normal mother	Likely benign VOUS
Pedigree 2	chrX:6538,033-7,100,181	562.1 kb	46, XX	*HDHD1,STS*	Inherited from normal mother	Likely benign VOUS
Pedigree 3	chrX:6,455,151-8,135,568	1.6 Mb	46, XX	*HDHD1,STS, PNPLA4,VCX*	Unknown	Likely benign VOUS
Pedigree 4	chrX:6,455,151-8,135,568	1.6 Mb	46, XX	*HDHD1,STS, PNPLA4,VCX*	Unknown	Likely benign VOUS
Pedigree 5	chrX:6,455,151-8,134,649	1.6 Mb	46, XX	*HDHD1,STS, PNPLA4,VCX*	Inherited from normal father	Likely benign VOUS

### Pregnancy outcome follow-ups

The clinical significance of the duplications in the fetuses was not clear. According to the database results and parental verifications, we considered that the duplications of Xp22.31 in our study were likely benign CNVs. Finally, as the follow-up shows, the pedigree 1 pregnant woman choose to terminate the pregnancy, as the baby girl was induced with no obvious abnormality; four pedigrees pregnant women choose to continue pregnancy, four female were born at full-term delivery. Follow-up results showed that the babies presented non-phenotypes at 3 months, 6 months and 1 year following their birth.

## Discussion

As the application of chromosome microarray technology advanced, increasingly more new pathogenic CNVs have been identified as being associated with disease. At present, there have been few studies on Xp22.31 microduplication, and the clinical significance of this duplication is still controversial. This has been reported in the healthy general population but also described in individuals with pathological conditions [13]. In our study, we report for the first time that five Pedigrees from Chinese population which were analyzed carried Xp22.31 microduplications The pathogenic effects of the duplication fragment were evaluated and genetic analysis was performed.

Xp22.31 microduplication usually includes genes *HDHD1, STS, PNPLA4, VCX* and *VCX3A* [1, 5–7]. Studies have shown that *STS* gene deletion is associated with XLI and that this gene duplication does not cause phenotypic abnormalities in males with recurrent duplications of Xp22.31 [16]. *HDHD1* encodes a member of the halogenated acid dehalogenase (HAD) hydrolase which belongs to a super family with unknown function [17]. *VCX* belongs to a family of genes with variable copy number on the X and Y chromosomes and is expressed only during spermatogenesis [18]. The *VCX* gene family consists of four genes on the Xp22.31 chromosome which include *VCX3A*, *VCX*, *VCX2* and *VCX3B*. In addition, *VCX3A* is expressed in the brain and is reported to be associated with abnormal neurocognitive phenotypes, and is described as a candidate gene in maintaining normal intelligence [7, 18–20]. The presence of the *VCX3A* gene in XLI patients of normal intelligence and its absence in XLI patients with intellectual disability suggest that a single intact copy of *VCX3A* is necessary to maintain normal intelligence levels [21]. Moreover, a VCX3A promoter deletion has been reported in a XLI case with borderline intellectual disability [22]. *PNPLA4* encodes for a member of the patatin-like phospholipase family involved in adipocyte triglyceride homeostasis and is essential for normal early development [23]. Li et al [5] found through a large cohort study that three consecutive cases of *STS* genes were found in healthy people, two of which were about 1.6 Mb, and the other which involved only the *STS* gene, with a carrier rate of 0.15% (3/2026). The affected sample carrying rate was 0.37% (29/7793), the ratio of male to female was 0.7 (12 males/17 females). In the same study, this duplication had not significant difference was found between the affected and healthy population (*p* = 0.1295). Liu et al. [1] proposed a possible neurobehavioral abnormality of Xp22.31 duplication. In the study, the prevalence in recurrent duplication was 0.289% (58/20095), the males or females prevalence is 0.226 or 0.382%, respectively. While, a total of 21 control individuals with the recurrent duplication were identified, for a prevalence of 0.41% (21/5088), the males or females prevalence is 0.182 or 0.523%, respectively. Moreover, the overall prevalence of the common recurrent duplication was not significantly different between patients and controls (*p* = 0.1573). Duplications of these genes do not appear to be significantly associated with intellectual disability or stunting. In addition, a recent study [24] found that, a proband with 600 kb in the Xp22.31 microduplication possessed hypotonia, malformation and developmental delay involving two genes, *VCX* and *PNPLA4*. This duplication was inherited from the external grandfather whose phenotype was normal. WES (whole exon sequencing, WES) revealed a previously reported de novo pathogenic mutation in *PURA* (Purine Rich Element Binding Protein A) in the proband that likely explains his severe phenotype. The expression of *PNPLA4* in Xp22.31 duplication was also studied, and the consequent increased RNA expression was detected in lymphoblasts from the proband as opposed to the controls [24]. However, PNPLA4 protein expression was comparable to in control males and females, which clarified that the gene *PNPLA4* escaped inactivation but the expression appeared tightly regulated at the protein level [24]. Therefore, the study suggests that the variable phenotype of the Xp22.31 microduplication may be due to the carrying of other mutations that are more severely affected the phenotype.

Our study shows that five pedigrees carried Xp22.31 microduplication in the Chinese population. According to the DECIPHER database, some cases smaller duplications showed pathological phenotype, some cases with larger duplication showed non-phenotypes. In addition, an evidence-based dosage-sensitivity map of the human genome showed that the *STS* ClinGen Haploinsufficiency Score 3, *STS* ClinGen Triplosensitivy Score 0, *HDHD1, VCX* and *PNPLA4* Haploinsufficiency/Triplosensitivy had not been evaluated. The duplications in the fetal chromosomes of the three pedigrees had been inherited from non-phenotypic parents, with two pedigrees refusing to perform parental verification. After full clinical consultation, four of the pedigrees continue their pregnancies. The follow-up results showed no abnormal clinical phenotypes. Considering the converging evidences in the role of the *VCX3A* gene in neuritogenesis, and negative evidence in an evidence-based dosage-sensitivity map of the human genome was proposed to be conducted with *STS* ClinGen Triplosensitivy. A study on the *VCX3A* gene revealed that this gene might play a critical role in the pathogenicity of the duplication event.

Through the clinical study of the five pedigrees after prenatal diagnosis, which carrying the Xp22.31 microduplications showed more likely to be benign CNVs. Follow-up results showed that the babies have non-phenotype, which were consistent with our interpretation of the duplication. Additionally, the parents’ verification showed that three fetuses were inherited from non-phenotype parents (two mothers and one father), which implied us those different sexes may have the same efficient and consistent with the previous study [1, 5]. Our research supports the idea that this duplication is a benign CNV [10, 11]. Although some studies showed that phenotypic abnormalities patients have a higher incidence of duplication than in healthy populations, however, there has no significant difference between the two groups. Moreover, recent studies have shown that the phenotypical diversity in the Xp22.31 duplication may be related to other genetic mutations performed by WES. Hence, from our investigation, it was believed that along with the popular of WES, some VOUS can provide new and meaningful findings; such that it might provide new insight into VOUS interpretation.

## Conclusions

Very few reports are available on Xp22.31 microduplication. This report is the first on Xp22.31 microduplication events in the Chinese population. Clinical and genetic investigation was performed on five different pedigrees carrying Xp22.31 microduplications. From our research, we consider that microduplications of these fragments are likely benign and had variable CNVs.

## Data Availability

The datasets used and/or analyzed during the current study available from the corresponding author on reasonable request.

## Abbreviations

Chr: Chromosome
CNVs: Copy number varitants
DGV: Database of genomic variants
OMIM: Online Mendelian inhertance in Man
SNP array: Single nucleotide polymorphism array
*STS*: Steroid sulfatase
*VCX*: Variable charge X-linked
VOUS: Variants of unknown significance
